# Effect of lysine supplementation on hypertensive men and women in selected peri-urban community in Ghana

**DOI:** 10.1186/s40795-017-0187-6

**Published:** 2017-07-27

**Authors:** Frederick Vuvor, Husein Mohammed, Thomas Ndanu, Obed Harrison

**Affiliations:** 1 Department of Nutrition and Food Science, School of Biological Sciences, College of Basic and Applied Sciences, University of Ghana, Accra, Ghana; 2 McGill University, Montreal, Canada; 3 Department of Community and Preventive Dentistry, School of Medicine and Dentistry, College of Health Sciences, University of Ghana Legon-Accra, Accra, Ghana

**Keywords:** Lysine, Hypertension, Placebo, Supplementation

## Abstract

**Background:**

Lysine is one of the essential amino acids and in cereal based diets it is known to be the most limiting and therefore determines the quality of dietary protein in such diets. This study investigated the effect of lysine supplementation on blood pressure of hypertensive adults in selected peri-urban community in Accra, Ghana.

**Methods:**

The study was a randomized double-blind controlled study made up of adults men and women assigned to a lysine supplemented group and a placebo-supplemented (control) group. The subjects aged between 18 – 45 years and totaling 180.

**Results:**

Total of 50 (28%) of the participating were hypertensive defined as Systolic Blood Pressure (SBP) ≥140 mmHg. The mean SBP of lysine-supplemented group significantly dropped from 146.11 ± 11.92 to 128.95 ± 10.44 mmHg (*p* = 0.02). That of women also dropped from 144.12 ± 10.41 to 132.28 ± 10.69 mmHg, (*p* = 0.06 while the control group had there SBP remained fairly constant after 112 days of intervention with changes in men from 145.79 ± 12.56 to 142.79 ± 11.07 mmHg (*p* = 0.32) and women in the control had very little drop from 145.15 ± 14.79 to 145.00 ± 17.93 mmHg (*p* = 0.96).

**Conclusion:**

Lysine supplementation resulted in normalization/reduction of blood pressure of hypertensive subjects who have suboptimal lysine intake.

## Background

There are about nine essential amino acids of which lysine is the most limiting, especially in cereal based foods and therefore affects the dietary protein quality. Studies have shown that dietary inadequacy of lysine leads to nonspecific indications of protein deficiency such as low resistance to disease, stress [1, 2]. The risk of lysine inadequacy is mostly found in regions where low socioeconomic groups depend on traditional roots, tubers and cereals for their protein supply [1, 2]. Many foods supply lysine, but the richest sources include red meats, fish, brewer’s yeast, and dairy products. Vegetables, on the other hand, are generally poor sources of lysine, with the exception of legumes. Evidence of the nutritional and health benefits of increased lysine content of cereal based foods is unlimited [3].

The majority of the deprived and undernourished populations in the world subsist on diets heavily based on cereals [4]. Such diets may probably be low in a number of essential nutrients including lysine. Comparisons about food availability data from various countries revealed that, there are significant decreases in the availability and affordability of animal-sourced foods and there is increased dependence on cereal-sourced foods. Study indicates that, lysine is the amino acid for which the largest differences occur between the diets of the rich and the poor [4].

Analysis conducted on data from 183 countries using food balance sheet has shown that there are high prevalence of lysine deficiency in most West African countries including Ghana [5]. Mean lysine in mg/g protein ranges from 43 mg/g protein to 34 mg/g protein. An average value below the adult male requirement of 45 mg/g protein indicates health risk for such populations. This is due to the fact that many of the most vulnerable are likely to have poorer quality diet (i.e. a greater dependence on cereal) [2]. The Ghanaian mean lysine was estimated to be 41.1 mg/g protein.

Though agricultural households in the peri-urban areas produce legumes and animals that could be used for household consumption, due to high market prices for these products, most households would prefer to sell them and purchase cheaper foods which are cereals, roots and tubers to feed their families [6]. The rate of malnutrition and morbidity are many times higher in peri-urban areas than in relatively more advantageous urban localities and even rural communities [6]. Animal and legumes protein that could supplement the amino acid pattern of cereals, are expensive for these groups [3].

Lysine is a constituent of a procedure proposed and implemented by Linus Pauling for the management of heart diseases. According to the outcome of his study, “when there are extra amount of lysine and proline in blood, the lipoprotein-(a) attachment sites get obstructed by lysine, creating a “Teflon-like” coating around the lipoprotein particles and hence prevents the lipoprotein-(a) from binding to the arterial walls. Therefore, lysine prevents plaque build-up and then initiates the setback of plaque deposits and invariably prevents pressure build up in the arteries and hence reduce hypertension” [7].

This lysine supplementation trial looked at the effects of lysine supplementation on blood pressure of hypertensive adults in selected peri urban community of Accra, Ghana. It was based on a hypothesis that supplementation of lysine in the diet of hypertensive subjects would significantly reduce or normalized their blood pressures.

## Methods

Sample size determination was done at the household level. This involved enrollment of equal number of men and woman pairs in each group. A total of 30 (15 were men and 15 women) in the placebo group and 30 (15 were men and 15 women) in the lysine group was arrived at. However, the sample size was increased to 45 males and 45 females to give 90 participants in each group making a total of 180 from 90 households containing one man and one woman from each household [4].

Exactly 428 households made up of 856 individuals were screened for eligibility. To be eligible, participants were supposed to be between the ages 18–45 years and should not be on any anti-hypertensive medications. Of these (428), 90 households totaling 180 individuals of 90 men and 90 women were recruited. Each household was randomly placed in the lysine or placebo group. Randomization was done at the household level to prevent supplements (lysine or placebo) from mixing between household members. The study was a double-blind and the supplements were identical in appearance.

The study protocol was approved by Ethical Review Board of Noguchi Memorial Institute for Medical Research (FWA 00001824-IRB000176). Informed consent form was signed by each recruited household head. The intervention (lysine supplemented) group consumed 1000 mg of lysine per day in two divided doses for a period of 112 continuous days.

Based on the calculations of Pellett [2], it is noted that at 41.1 mg lysine /g protein, lysine availability in Ghanaian diets is low. Table 1 shows the supplementary lysine required to complete the protein quality. An extra 15.2 mg lysine/g protein or 900 mg of lysine per day was needed to supplement the diets of Ghanaians. The lysine was provided in tablet form. Each subject was supplied two tablets of lysine-HCl (each tablet equivalent to 500 mg lysine per tablet or 500 mg of di-calcium phosphate as a placebo) per day for 112 days. Supplements were formulated by Disto Pharmaceuticals, in Hyderabad.

**Table 1:** Lysine requirement and addition calculation for Ghana

	Unit per capita
Lysine^a^	mg/ day = 2240
Lysine^a^	mg/g = 41.1
Current recommended lysine value	mg/g protein = 45.0
Addition 25%	Buffer = 56.3
Lysine to be added	mg/g = 15.2
Total lysine needed	mg/day = 826
Total Lysine to be added	mg per day = 908
Total Lysine HCL	mg per day = 1135

^a^Source: [5] FAO/Faostat (2004). Data for 2001. (Adapted from Pellett 2004)

Lysine mg/g Protein from the formula: *Lysine values in milligrams per day were calculated from the equation: Lysine = (86.3 × APg/day) + (19.8 × CPg/day) + (63.6×PSg/day) + 599 where AP, CP and PS were animal, cereal and pulse-soy protein respectively. Lysine values in milligrams per gram of protein were derived by further dividing by the amount of total protein* [5] FAO/FAOSTAT (2004) (Adapted from Pellett 2004).

Information collected at starting point included: dietary, anthropometry and blood pressure (BP) measurements. A repeated 24-h dietary recall was done for 3 non-consecutive days for each subject including one weekend to obtain a representative data on dietary intake. For the 24 h recall, the participants were asked to mention all the foods they had consumed in the past 24 h prior to the interview. With the help of food models they were made to estimate the quantities or portions they consumed and with the help of food composition tables, their nutrient intake was estimated Blood plasma lysine is known to be poor indicator of dietary lysine status thus only dietary information was used to determine lysine levels [8].

Dietary data were converted into nutrient content using Ghanaian Food Composition Tables and [9] supplemented by US Department of Agriculture Table [10], owing to absence of amino acid data in the ‘Ghanaian Food Composition Tables’. Nutrient examination included type of protein (animal, legume, vegetable and roots and tubers), utilizable protein levels based on the PDCAAS (*Protein Digestibility Corrected Amino Acid Score*) method and expressed as total utilizable protein per day and utilizable protein g/kg body weight, lysine, sulphur amino acids, threonine and tryptophan (mg/day and mg/g protein), total energy, carbohydrate and fat.

Adequacy of nutrients intake was assessed using FAO/WHO Recommended Dietary Allowance (RDA) and Estimated Average Requirements (EAR) [5]. Adequacy of nutrient intake were determined employing the EAR cut-point method [11] to evaluate the nutrients adequacy of subjects. The population prevalence of inadequacy of a given nutrient is the proportion of the population with intakes below the EAR [12]. Nutrients intakes were compared to the RDAs for each subject. Protein intakes were categorized by sources namely cereal protein, legume protein, animal protein, protein from fruits and vegetables, protein from roots and tubers, and protein from other plant sources. Energy intakes were expressed as the percentage contributions from the various sources namely carbohydrate, protein and fats.

All the anthropometric measurements including weight and height were collected using standard procedures. Weight and height were taken using the weighing scale and Stadiometer respectively. Body Mass Index (BMI) was calculated as weight in kilograms divided by the square of height in meters. Clinical assessments involved the determination of BP before and after supplementation/intervention. Blood pressure was recorded using a digital sphygmomanometer. Here, the participants were made to relax and sit comfortably. It was made sure that participants had recently emptied their bladder prior to the measurement. Tight sleeved clothing were removed and lose ones were rolled up. They were made to rest in a chair next to a table for about 5–10 min. Their left arm was gently positioned at heart level with palm facing up. Seated up straight with legs uncrossed and forearm on the table, the arm cuff was wrapped around it. The start button of the sphygmomanometer was pressed and the reading were recorded with alongside with the date and time after the automated process. All measurements were done in triplicates and their averages were used. A subject was classified as hypertensive if SBP ≥140 mmHg (systolic BP). The design of the monitoring was such that the tablets were provided on a weekly basis during the intervention period.

Epi-Info 2000, MS Excel 2007 and SPSS version 16 were used for data analyses. Statistical analysis included comparisons between groups for differences in diets, anthropometry and blood pressure. Data analysis was carried out using paired and independent t-test for comparison of measured parameters followed by analysis of variance (ANOVA). Changes in BP (Delta) values were calculated for both men and women groups (lysine and placebo). The chi-square test was used for comparison of categorical variables between the two treatment groups. Significance level was set at 0.05. An unpaired t-test was employed to compare the lysine-supplemented and the placebo-supplemented subjects. The paired t-test was used to compare the delta of baseline and posttest data of all categories (normotensive, and hypertensive).

## Results

### Background characteristics

More than 50% of the participating men were artisans, while more than 60% of the women were food vendors.

### The baseline characteristics

Table 2 shows that, at the start of the study all subject and conditions were fairly similar and comparable.

**Table 2:** Characteristics of subjects at baseline

Variable	Men		Women	
	Lysine^a,b^ (*n* = 46)	Placebo1, 4 (*n* = 44)	Lysine1,4 (*n* = 46)	Placebo1,4 (*n* = 44)
Age y	33.2 ± 8.2	33.5 ± 7.7	31.4 ± 7.0	32.2 ± 6.6
Body Mass Index kg/m2	22.60 ± 3.41	22.43 ± 3.09	25.67 ± 5.28	27.64 ± 5.76

^a^Mean ± SD (all such values). ^b^None of the values were significantly different at *p* ≤ 0.05

#### Dietary intakes among the study groups

Examining the differences in habitual dietary intakes by treatment type showed that there was no differences in energy, total and utilizable protein, and lysine (in both mg/kg body weight and mg/g protein) among all the groups. Adding of 1000 mg lysine accounted for 0.19 g of N which accounted for 2.3% in men, 2.7% in women based on their habitual intake (Table 3). This percentage increase did not significantly add to the overall nitrogen in the lysine versus control group (Table 3); but led to a reduction in risk of protein and lysine inadequacy in the group (Table 3).

**Table 3:** Dietary intakes among the study groups at baseline

	Lysine group1			Lysine group +1000 mg lysine2			Placebo group1		
	*Mean*	*SD*	*% Risk of inadequacy3*	*mean*	SD	*% Risk of inadequacy3*	*Mean*	*SD*	*% Risk of inadequacy3*
Men	*n = 46*			*n = 46*			*n = 44*		
Total Energy kcal	1709	386		1709	386		1772	399	
Total Protein g	51.35	14.51		51.35	14.51		53.37	13.35	
Total N g	8.22	2.32		8.41	2.32		8.18	2.28	
Utilizable Protein g 3	49.95	15.32	38.4	51.35	14.51	33.5	51.15	14.25	35.8
Lysine mg/kg/day 4	38.55	13.08	32.6	53.86	13.71	0	41.54	19.45	29.5
Sulfur Amino Acids mg/kg/day 4	23.49	6.93	10.9	23.49	6.93	10.9	24.74	9.22	11.4
Women	*n = 44*			*n = 44*			*n = 41*		
Total Energy kcal	1406	483		1406	483		1435	335	
Total Protein g	42.88	13.75		42.88	13.75		45.05	13.80	
Total N g	6.86	2.2		7.05	2.20		6.98	2.25	
Utilizable Protein g3	40.82	13.65	56.6	42.87	13.76	51.2	43.65	14.10	58.7
Lysine mg/kg/day 4	32.20	12.34	50	48.41	13.99	11.4	33.44	16.94	48.8
Sulfur Amino Acids mg/kg/day4	20.00	7.43	31.8	20.00	7.43	31.8	19.69	8.23	39.0

No significant differences in the lysine and control groups before adding lysine tested using independent sample t-test

The group reveals the expected enhancement in baseline value of the lysine group nutrients on adding 1000 mg of lysine which is corresponding to the quantity of supplement supplied

Percent at risk of protein deficiency, based on a requirement of protein of 0.66 g/kg intended for adults (WHO, 2007)

Adult amino acid requirements: 30 mg/kg/day lysine, 15 mg/kg/day sulfur amino acids, 15 mg/kg/day threonine, 4 mg/kg/day tryptophan (WHO, 2007)

Difference from placebo group, *p* < 0.001

In some other parts of the developing world, cereals have been reported as the major source of dietary protein; an example is rural Bangladesh where cereals contribute up to 53% of total protein intake [13]. The proportions of proteins contributed by the different food groups have implications on the protein quality. Animal proteins are known to be of a higher quality than plant proteins because they contain all the essential amino acids unlike plants foods which do not.

Examining the amino acid balance (Table 4) in comparison with ‘WHO reference pattern’ revealed that, before the supplementation, women and men in the placebo and lysine group met all amino acids (total sulfur amino acids, threonine and tryptophan) reference pattern [14] but not the lysine. Adding lysine enhanced the lysine score but it did not make any other amino acid limiting which is a function of amino acid score.

**Table 4:** The amino acid balance in comparison to WHO 2007 reference and on the addition of 1000 mg of supplement at baseline

	Lysine1			Lysine + 1000 mg lysine			Placebo1		
	*Mean*	*SD*	*Percent below reference pattern*	*mean*	SD	*Percent below reference pattern*	*Mean*	*SD*	*Percent below reference pattern*
Men	*n = 46*			*n = 46*			*n = 44*		
Lysine mg/g protein 3	55.59	7.76	10.9	79.47 2	9.87	0	55.78	9.57	13.6
Sulfur Amino Acids mg/g protein3	34.12	2.84	0	34.12	2.84	0	33.76	3.16	0
Women	*n = 44*			*n = 44*			*n = 41*		
Lysine mg/g protein 3	53.01	7.29	15.9	81.962	11.15	0	55.28	9.04	9.8
Sulfur Amino Acids mg/g protein3	32.82	3.31	0	32.82	3.31	0	32.99	3.36	0

No significant differences between dietary intakes in the lysine and control groups before adding lysine tested using independent sample t-tests

This group reflects the expected improvement in baseline nutrient value of the lysine group diets upon the addition of 1000 mg of lysine which is equivalent to the amount of supplement provided

Adult amino acid reference pattern: 45 mg/g protein lysine, 22 mg/g protein sulfur amino acids, 23 mg/g protein threonine, 6 mg/g protein tryptophanTable 5: Effects of lysine supplementation on the BP of hypertensive participants enrolled in the study

#### Blood pressure

At baseline, 41% of males and 17% of females in the lysine group were hypertensive whereas in the placebo group, 43% of males and 29% of females were hypertensive. There was no significant difference between the lysine and placebo groups at baseline. Generally lysine supplementation resulted in a reduction in SBP of hypertensive subjects. The mean changes in SBP levels of subjects at baseline and at post supplementation were compared. Hypertensive was defined as SBP ≥140 mmHg. The results in Table 5 shows a reduction /normalization of SBP in lysine-supplemented hypertensive groups but not in the control groups. Also no significant change was observed in men and women who were normotensives (SBP < 140 mmHg).

**Table 5:** Effects of lysine supplementation on the BP of hypertensive participants enrolled in the study

	Lysine1			*p-value*	Control1			*p-value*
	Baseline	Posttest	Change		Baseline	Posttest	Change	
Men								
n3	19	19	19		19	19	19	
Systolic (mmHg)	146.11 ± 11.932	128.95 ± 10.44	7.16 ± 12.65*	0.02	145.79 ± 12.56	142.79 ± 11.07	3.00 ± 12.87	0.32
Women								
n3	8	8	8		13	13	13	
Systolic (mmHg)	144.12 ± 10.41	132.38 ± 10.69	5.75 ± 7.02	0.06	145.15 ± 14.79	145.00 ± 17.93	0.15 ± 11.48	0.96

Paired-sample t tests: Mean ± SD (all such values): Sample size: *P < 0.05

## Discussion

The greatest proportion of household heads (38%) were artisans. This was in line with the fact that several construction works were going on in the study area, thus creating job opportunities for carpenters, masons and the like. The study indicates that energy intake was generally low among study participants with all age group having a mean energy intake below the RDA. Meeting energy requirements has been a problem in developing countries. The fact still remains that over 300 million people in Africa consume less than 2100 cal per day on average [10].

On the contrary protein intake was found to be quite adequate among study participants. About 70% of subjects met their EAR for proteins and all age groups had average intake values >60% of the RDA. This may be attributed to the availability of animal sourced foods, particularly fish, meat and eggs in the study area. This observation is contrary to expectation since protein deficiency is one major nutritional problem in Ghana. It however implies that nutritional problems in the country differ from community to community and thus intervention programmes should be based on findings in different communities. Among women however, a high proportion (47.7%) had inadequate protein intakes (intake less than their EAR). There is also the likelihood that some subject over-reported their intake of protein-rich foods since frequent consumption of such foods including fish, meat and eggs are seen as an indicator of wealth.

**Fig. 1:**
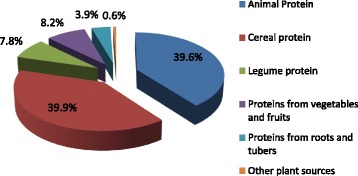
Shows the percentage distribution of different food groups to protein intake among the participants in the study. Approximately 40% of the protein intake came from cereals. Animal source foods contributed about 40% dietary protein intake; legumes contributed just about 8%

**Fig. 2:**
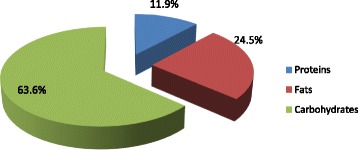
displays the contributions of carbohydrate, protein and fats intake among study participants. Protein contributed about 12%; carbohydrate about 64%; and fats contributed 25% of energy intake

Our findings further showed that nearly equal proportions of total protein intakes came from cereals and animal foods (Fig. 1). Main animal foods found in the diets of subjects were fishes, although meats and eggs were also present in significant amounts. This confirms that among people living in the coastal regions, fish constitutes about 60% of animal protein intake (National Fisheries Association of Ghana, 2004). The high proportion of proteins from cereals may be attributed to the frequent consumption of these foods and their products in the study population. Cereal products such as maize and rice, were consumed nearly daily by study participants (Fig. 2). Thus protein quality among study participants can be said to be quite adequate. Lysine is the first limiting amino acid in nearly all underdeveloped countries and certainly, about 30% of men and 50% of women are deficient of lysine (mg/kg/day) [15, 16].

Energy intake among subjects was generally inadequate. Hypertension was higher in males than females (*p* = 0.023). Using the paired sample t-test our data shows that the lysine supplementation resulted in a significant reduction in BP of hypertensive men (*P* = 0.024) but there was no other changes in BP in both men and women hypertensive among the control group.

After 112 days of intervention lysine-supplemented group recorded a significantly lower variation in SBP than the placebo supplemented group. Results from Hussain et al.*,* (2004) and Smriga et al.*,* (2004) [3, 4] have shown that some stress responses in low economic populations consuming mainly cereal diets can be enhanced with lysine supplementation.

About 28% of the participating subjects were hypertensive. The mean SBP of hypertensive men significantly dropped from 146.11 ± 11.93 to 128.95 ± 10.44 mmHg (*p* = 0.02), while the women dropped from144.12 ± 10.41 to 132.38 ± 10.69 mmHg (*p* = 0.06). The change in men on control was from 145.79 ± 12.56 to 142.79 ± 11.07 mmHg (*p* = 0.32) and women on control from145.15 ± 14.79 to 145.00 ± 17.93 mmHg (*p* = 0.96). This study revealed that adequate lysine intake could significantly reduce the BP of hypertensive patients especially those on low lysine diets.

The granular neurons in the cerebral cortex of the brain are of two types, one functions as excitatory while other as inhibitory neurotransmitters mainly [17]. Glutamate serves as excitatory and gamma-aminobutyric acid (GABA) functions as inhibitory neurotransmitters [17]. Lysine is directly involved in the synthesis of GABA and which reduces stress and anxiety [17]. The deficiency of lysine reduces the availability of GABA. It stands to reason that there is a strong relationship between diets low in lysine and high blood pressure which may be due to excess stress/anxiety as a result of low levels of lysine intake.

### Strengths and limitations

This study had several strengths regarding the potential benefit of lysine supplement on improving hypertension; however, other factors related to blood pressure had not been controlled such as whether there were any changes in dietary intakes of the 112 days and also other lifestyle factors. This is a limitation that can be improved upon for further studies.

## Conclusions

The results suggest several possible protective effects of lysine on hypertension. Lysine could directly help reduce BP of hypertensive individuals whose lysine intakes are low. Lysine could also have control over the abnormal responses to stressors/anxiety which is known to influence heart beats and therefore on hypertension. After 112 days of daily intake of 1000 mg of lysine, lysine-supplemented group recorded a significant reduction in SBP compared with the control hypertensive group. It could be concluded that adequate lysine intake may have possible positive effects on blood pressure of individuals with no underlying secondary causes. These results also suggest that lysine may be a useful nutrient adjunct to hypertension medications.

## Abbreviations

ANOVA: Analysis of variance
AP: Animal protein
BP: Blood pressure
CP: Cereal protein
EAR: Estimated average requirements
FAO: Food and agriculture organization
GABA: Gamma-aminobutyric acid
PDCAAS: Protein Digestibility Corrected Amino Acid Score
PS: pulse-soy protein
RDA: Recommended dietary allowance
SBP: Systolic blood pressure
SPSS: Statistic package for social sciences
WHO: World health organization
